# Window into the mind: Advanced handheld spectroscopic eye-safe technology for point-of-care neurodiagnostic

**DOI:** 10.1126/sciadv.adg5431

**Published:** 2023-11-15

**Authors:** Carl Banbury, Georgia Harris, Michael Clancy, Richard J. Blanch, Jonathan James Stanley Rickard, Pola Goldberg Oppenheimer

**Affiliations:** ^1^ School of Chemical Engineering, Advanced Nanomaterials Structures and Applications Laboratories, College of Engineering and Physical Sciences, University of Birmingham, Edgbaston, Birmingham, B15 2TT, UK.; ^2^ Ministry of Justice, 102 Petty France, Westminster, London, UK.; ^3^ Department of Military Surgery and Trauma, Royal Centre for Defence Medicine, Birmingham, UK.; ^4^ Neuroscience and Ophthalmology, Institute of Inflammation and Ageing, College of Medical and Dental Sciences, Robert Aiken Institute for Clinical Research, University of Birmingham, Edgbaston, Birmingham, B15 2TT, UK.; ^5^ Department of Ophthalmology, Queen Elizabeth Hospital Birmingham, UHB NHS Foundation Trust, West Midlands, UK.; ^6^ Department of Physics, Cavendish Laboratory, University of Cambridge, JJ Thomson Avenue, Cambridge, CB3 0HE, UK.; ^7^ Healthcare Technologies Institute, Institute of Translational Medicine, Mindelsohn Way, Birmingham, B15 2TH, UK.

## Abstract

Traumatic brain injury (TBI), a major cause of morbidity and mortality worldwide, is hard to diagnose at the point of care with patients often exhibiting no clinical symptoms. There is an urgent need for rapid point-of-care diagnostics to enable timely intervention. We have developed a technology for rapid acquisition of molecular fingerprints of TBI biochemistry to safely measure proxies for cerebral injury through the eye, providing a path toward noninvasive point-of-care neurodiagnostics using simultaneous Raman spectroscopy and fundus imaging of the neuroretina. Detection of endogenous neuromarkers in porcine eyes’ posterior revealed enhancement of high–wave number bands, clearly distinguishing TBI and healthy cohorts, classified via artificial neural network algorithm for automated data interpretation. Clinically, translating into reduced specialist support, this markedly improves the speed of diagnosis. Designed as a hand-held cost-effective technology, it can allow clinicians to rapidly assess TBI at the point of care and identify long-term changes in brain biochemistry in acute or chronic neurodiseases.

## Teaser

The developed portable Raman device technology detects neurochemical changes following traumatic brain injury via the eye.

## INTRODUCTION

Traumatic brain injury (TBI) has become a leading clinical challenge of the 21st century affecting 135 million people globally. TBI injuries evolve immediately after the initial trauma, yet many individuals display very few clinical symptoms at the early stages, which, however subsequently, develop long-term, persistent, neurodegenerative deficits (*1*–*3*). As the brain tissue lacks regenerative capacity, early diagnosis is crucial for improving outcomes. Life-critical decisions, which influence patients’ prognoses and the efficacy of treatment, must be made within the first hour after trauma (i.e., the “golden hour”). Hospital radiological investigations, involving computer tomography (CT) or magnetic resonance imaging, are expensive and cannot be used in a timely manner at the point of care, where the assessment by ambulance crews still relies on blunt observational triage tools with macro-descriptors transmitted via third-party intermediaries to clinicians. The Glasgow Coma Scale (GCS) is widely used to predict patients’ outcomes; however, this has limitations of variability of inter-rater reliability, predictive validity, inability to evaluate the verbal part of measure for endotracheally intubated patients, obscuration by sedation, and the inability to detect small changes in TBIs, which can be present without any detectable physiological abnormalities. It is known that in TBI misdiagnosis or delay in treatment in the prehospital settings is where most of the acute, cerebral damage occurs. Now, no point-of-care technology exists for quantitative assessment of TBI with sufficient sensitivity and timeliness to aid the stratification and early diagnosis whether this is at the pitch side in contact sports or the roadside after motor vehicle collisions. This is exacerbated by the long-term consequences of mild TBI and concussion, with cumulative effects from multiple sustained injuries for athletes and the military.

To address the challenges associated with early-stage detection of TBI, we have developed an unconventional laser-based spectroscopic technology focused on analyzing the neuroretina and optic nerve at the back of the eye, as a projection of brain tissue. This structure, bathed in cerebrospinal fluid and in continuity with the central nervous system (CNS), provides an optically clear window into the biochemistry of the brain (*4*–*8*). By targeting CNS biochemical changes, we reduce the need to filter out confounding (non-CNS) compounds, measuring the brain side of the blood-brain barrier.

At the back of the eye exists a small part of the brain covered only by optically clear media, the retina containing all retinal ganglion cell bodies and the optic disc, through which all retinal ganglion cell axons leave the eye, carrying visual information captured by the retina to the brain through the optic nerve. Ganglion cells in the retina are unmyelinated, meaning that changes assessed there relate directly to neuronal biology, while the optic nerve is myelinated and surrounded by cerebrospinal fluid, continuous with that surrounding the rest of the CNS. The retina and optic nerve have long been known to display physically measurable changes because of increased intracranial pressure, where its monitoring is of significance for intensive care in TBI (*9*–*12*). To interrogate these eye layers, we have developed an eye-safe device (EyeD), based on using multiplex resonance Raman spectroscopy, targeted at specific TBI biomarkers or “molecular fingerprints,” as proxies for disease and injury. Such markers for “brain health” include structural changes in brain-specific lipids and biochemicals due to local tissue damage such as cardiolipin and cytochrome C or multiple specific neuromarkers such as S100B, glial fibrillary acidic protein (GFAP), or the *N*-acetylasparate, found exclusively in the CNS. In the acute TBI phase, these have demonstrated strong correlation with injury severity, relating to radiological, surgical, and physiological findings (*13*–*17*).

**Fig. 1.**
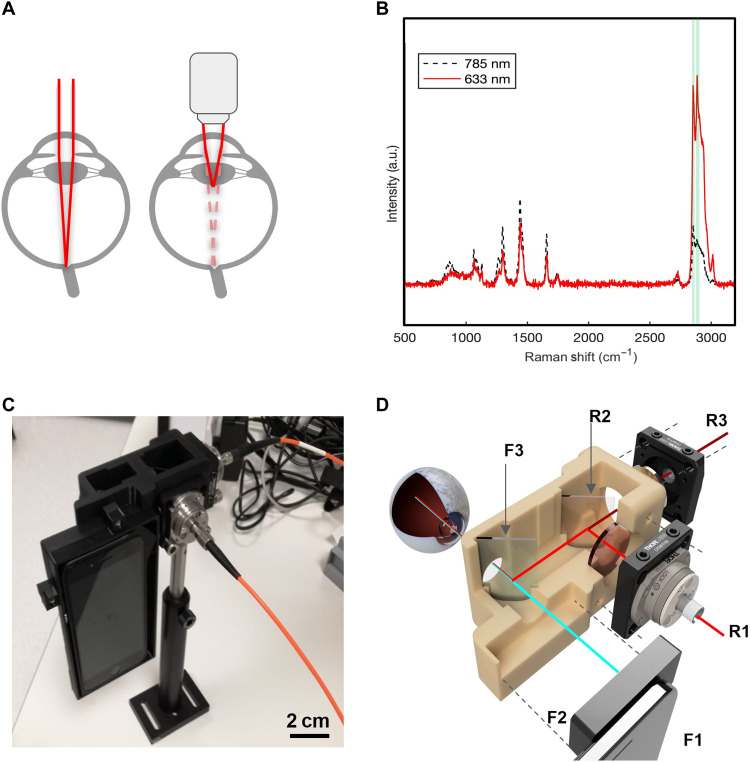
The engineered EyeD overview. (**A**) Convergence of a collimated beam entering the eye onto the retina (left) and the compound lens effect (right) resulting from the introduction of a microscope objective. (**B**) Representative Raman spectra of murine brain tissue in the fingerprint and high–wave number regions, measured using a commercial In-Via Raman, with an excitation laser of 633 nm (0.39 to 0.63 mW). (**C**) 3D schematics of the combined fundus photography and eye-safe Raman spectroscopy optical paths contained within a 3D-printed housing. (**D**) Photograph of the bench-top breadboard setup, including smartphone, housing, and input/output fibers. a.u., arbitrary units.

Concurrently, Raman spectroscopy is a highly specific analytical technique and rapid, which can provide real-time, quantitative diagnostic information in clinical settings both in vivo and ex vivo by measuring subtle changes to inelastically scattered light, accurately identifying changes in disease-specific biomarkers with diagnostic capabilities (*18*–*21*). In ophthalmology, Raman spectroscopy has been applied to study disease states ex vivo (*22*–*24*). Further findings highlighted the ability to identify malignant tissue in the brain, acting as a surgical guide. However, these required direct and invasive access to the brain (*18*). We have recently demonstrated a path toward the detection of changes to brain chemistry by acquiring spectra from tissues of the retina to identify TBI in ex vivo murine model and established a high-accuracy differentiation of degrees of brain injury severity using a commercial laboratory-based Raman spectroscopy (*17*). We have further demonstrated that a range of validated CNS biomarkers, rapidly and continuously released from injured neurons into the blood, were detectable in cerebrospinal fluid, reflecting biochemical changes occurring in the brain and creating temporal profiles of extracellular activity post-TBI (*16*). TBI was subsequently classified from spectral data on mice by detecting tissue-specific signatures of each anatomical layer on eye sections using a commercial Raman system (*17*, *25*). Marro *et al.* and Stiebing *et al.* (*26*, *27*) have studied the retina using a standard Raman arrangement and flat mounted or cultured tissue. However, the major obstacle to in vivo imaging has been the use of high-magnification, high–numerical aperture (NA) objective lenses, which are typically required for Raman spectroscopy. Such objectives have a natural incompatibility with imaging the eye posterior since the eye itself can be considered as a lens with a combined positive power of 60 diopters (*28*). Thus, the combined eye-microscope optics led to a compound lens arrangement, which shortened the working distance of the microscope lens (Fig. 1A).

**Fig. 2.**
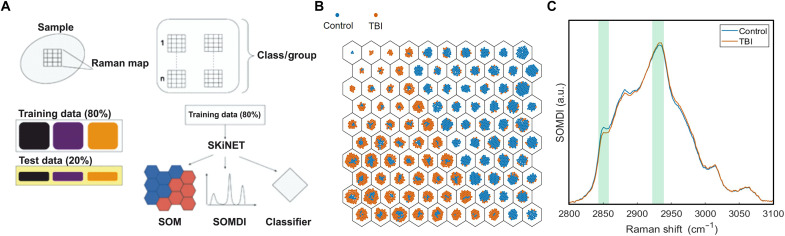
Illustration of data analysis workflow via SKiNET with representative classification outputs via SOM and SOMDI. (**A**) In this workflow, spectra measured from Raman maps are grouped according to class or group studied. A 20% partition of the data is randomly selected and reserved as test data. The remaining 80% is input into SKiNET, which directly provides dimensionality reduction (SOM), SOMDI feature extraction and classification. SKiNET is optimized on the training data using cross-validation and adjusting the available parameters (number of neurons, initial learning rate, and number of training steps) to maximize the classification accuracy on the training data. Last, the optimized model is shown the previously unused test data and asked to classify each spectrum as either TBI, control, or the various brain injury severity subgroups. (**B**) SOM spatial clustering of high–wave number spectra from control and TBI tissue of the murine retina. (**C**) SOMDI extracted Raman features distinguishing TBI and healthy control [determined by one-way analysis of variance (ANOVA)] (*P* = 0.0090) groups from the corresponding SOM in (B).

Here, we have developed and engineered an unprecedented portable and noninvasive EyeD technology, without using ionizing radiation, combining modified optics with fundus imaging of the optic nerve together with a class I laser introduced into the optical path and focused by the eye. Our unique device permits simultaneous Raman spectroscopy and fundus imaging by isolating the Raman and white light paths. Via the EyeD, Raman signals are collected using a detector, and data are classified using the developed artificial neural network algorithm as a decision support tool (*29*) and the self-optimizing Kohonen index network (SKiNET) as a framework for an advanced multivariate analysis (*30*–*36*), which simultaneously provides (i) dimensionality reduction, (ii) feature extraction, and (iii) multiclass classification (Fig. 2A). SKiNET performs visual separation to identify the underlying chemical differences between classes, providing accurate classification for simultaneously rich-information and high-classification specificity, even for low laser powers and short acquisition times, representative of the real-world point-of-care conditions. SKiNET’s intrinsic self-optimizing maps (SOMs) provide visually intuitive two-dimensional (2D) clustering, i.e., according to injury state, of high-dimensional spectral data, which are otherwise difficult to interpret for large sample numbers. SKiNET incorporates supervised learning to additionally provide accurate classification, which could then be used to make diagnostic predictions. Further, self-optimizing map discriminant index (SOMDI) feature extraction identifies which spectral features, i.e., chemical changes, are responsible for the clustering seen in SOMs. Applying the EyeD, integrated with SKiNET, to investigate the retina and the optic nerve, reflecting the brain environment after injury, rapidly distinguishes TBI from control groups, yielding an automated classification of the acquired Raman data and assignment to the particular neuromarker, tissue type, or disease state. This tool, along with the successful demonstration that high-frequency Raman bands indicative of early-stage TBI, can be safely measured from the neuroretina and the optic nerve, enabling important steps for translation of the developed platform technology to real-world clinical point-of-care neurological diagnostic applications.

Rapid, portable EyeD is designed for use on-site for immediate decision-making and treatment. Measuring abnormal changes in the optic nerve at the point of care would be indicative of TBI, providing a quantitative assessment of trauma at the earliest stages while simultaneously helping to quantify the damage. It would be interpreted by clinicians as an indication to treat the patients according to TBI guidance without delay and help in triaging, e.g., directing to major trauma centers with neurosurgical facilities. Neuroprotective measures would be instigated immediately irrespective of the exact diagnosis (a more detailed pathoanatomical classification would come later, after in-hospital neuroimaging).

## RESULTS AND DISCUSSION

As a result of the fundamental restriction imposed by the optics of the eye shown in Fig. 1A, we have acquired the Raman spectra from the retina using a collimated beam incident on the cornea, allowing the eye to naturally focus the beam onto the retina. This has previously been limited to the identification of age-related macular degeneration, by exploiting resonance Raman of macular pigments (*37*). Such an effect markedly enhances the available signal, which helps to mitigate the restricted laser power and absence of high-power optics.

Figure 1B shows the representative Raman spectra of murine brain tissue measured using an excitation wavelength of 633 nm spanning the fingerprint and high-frequency regions, from a commercial Raman instrument (Qontor InVia Renishaw). We observe an apparent enhancement of high–wave number bands, associated with the resonance of the methylene overtone at 619.68 nm (*38*), which normally yield relatively weak peaks with the 785-nm excitation. In addition to the strong high–wave number response, these bands suffer little interference from fluorescence, which often tends to dominate the fingerprint region. Further to the detected enhanced response from the high–wave number region of the murine tissue identifying the bands, which alone are capable of detecting the presence of TBI, an eye safe 635-nm class I laser, guaranteeing eye safety, has been identified. The use of fiber optics allows us the design and engineering freedom, allowing for bulky components to be kept away from the patient, ensuring a compact imaging system. Silica used in fiber optics normally yields additional interference due to its own Raman signal in the fingerprint region. However, in the high–wave number region, there is no Raman contribution from silica. Via the combined ability to avoid interference from fluorescence as well as Raman scattering from optical fibers, we have established a suitable spectroscopic portable setup enabling the detection of high–wave number Raman bands from the retina. The overall engineered EyeD consists of the combined fundus imaging optical path and eye-safe Raman spectroscopy path contained within a 3D-printed housing (Fig. 1, C and D).

The design that houses the optics is based on a split casing and an indented lid to overlap the housing and lid components when secured, limiting external light entering the system. Optical components fit into a cradle and allow for easy adjustments and optimization in free space with an open-on-top section for alignment during optimization (figs. S2 and S3). The fundus imaging path consists of a smartphone (F1), D-EYE fundus module (F2), 625-nm short-pass dichroic beam splitter (F3), and the eye (F4). The Raman spectroscopy path consists primarily of a 635-nm class I laser (R1), 635-nm dichroic beam splitter (R2), and spectrometer (R3), converging with the imaging path at F3.

Previously, we have shown that Raman spectroscopy can be used to detect mild TBI from the retina (and brain) in a murine model using the 785-nm excitation laser and formed a classification model (*17*, *25*, *29*) using a commercial Raman spectrometer in the fingerprint region. High–wave number measurements recorded from these murine tissue samples display a clear separation between healthy controls and TBI groups via SOMs (Fig. 2B) with a subtle but clear change in the ratio of the bands around 2850 and 2930 cm^−1^ observed from features extracted using SOMDI to rapidly detect the TBI cohorts (Fig. 2C).

The acquired data are classified using the artificial neural network algorithm, SKiNET as a decision support tool, based on SOM with a classification via SOMDI. An illustration of the workflow is shown in Fig. 2A. Through inspection of key differences between neuron weights and class weight vectors, the algorithm enables identification of the key spectral changes. These allow the identification of the types of data a given neuron activates, which are then used to inspect the weights across all neurons and extract prominent features belonging to each class by finding the weights that contribute most to a particular class. The peaks in SOMDI subsequently correspond to cm^−1^ and modes that contribute most to the clustering observed in the SOM. Training parameters used for the SOM include grid size, learning rate, and optimal number of epochs, and the separation of classes reveals the characteristic differences due to the classification of certain neurons. This enables a clear basis for differentiation via the characteristic weight vectors to be derived in SOMDI.

SKiNET is based on the separation of data classes in a SOM, which loosely mimics the visual cortex in the brain with the neighboring neurons activating on similar inputs, and the undefining characterization using a SOMDI, enabling the rapid subsequent classification of the tested data. SOM defines 2D maps of neurons, typically arranged as a grid of hexagons. Each neuron is assigned a weight vector, which is initialized randomly and has a length equal to the number of variables in a spectrum. The weight vector affects which neuron is activated for a given sample with the neighboring neurons having similar weights. Spatial clustering is therefore observed in the trained SOM, with spectra that exhibit distinct properties activating different neurons. To extract the information on which spectral features are responsible for certain neurons activating over others, a SOMDI is used. SOMDI provides a representation of weights associated with neurons identifying a particular class by introducing class vectors as labels for each spectrum and corresponding weight vectors for each neuron, without influencing the training process, allowing the identification of what type of data a given neuron activates, used to inspect the weights across all neurons and extract prominent features belonging to each class. Neurons (hexagons) are colored according to the modal class they activate, from the Raman spectra and those that have no majority class or activate none of the data are colored as white. For each class, there is a clearly defined block of neurons, with many of these activating only a single tissue or biomarker type and a higher SOMDI intensity indicates a greater importance of wave number. Further, by inputting a test sample into the trained neural network and detecting which neuron has been activated, the associated SOMDI provides class data, which is then used to make a prediction for the unseen sample, enabling SKiNET to be used as a classifier.

Colored circles within each neuron represent spectra from the training data that have been activated for that neuron. To aid visualization, circles are forced to not overlap in space using the D3force library, providing an alternative mechanism to display sample frequency and class overlap for each neuron. For each class, there is a clearly defined block of neurons, with many of these activating only a single tissue type. An approximately even distribution in the number of neurons required to identify each class is observed. The SOMDI provides a representation of weights associated with neurons that identify a particular class. A higher SOMDI intensity indicates greater importance of particular inverse centimeters along the axis of a spectrum. This, despite the level of overlap or noise in the original data, enables well-defined peaks to be resolved, which are either more prominent or unique to each class.

**Fig. 3.**
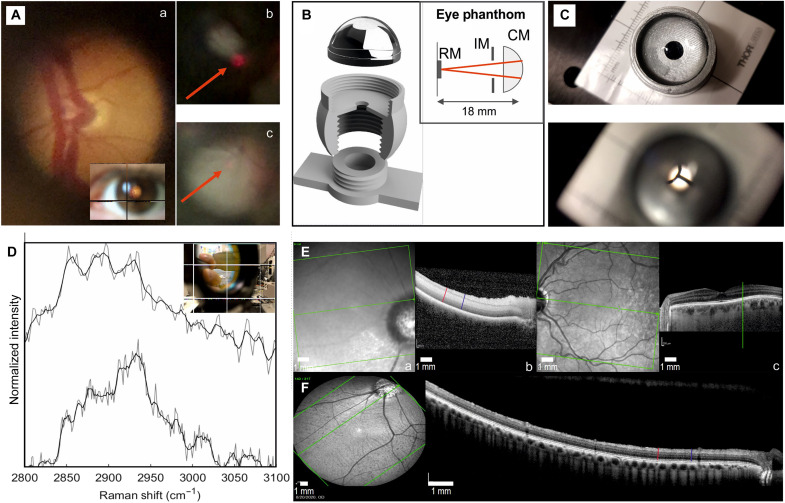
Eye phantom, fundus imaging with the coaligned system, and the OCT. (**A**) (a) Fundus image of a human eye acquired using an unmodified D-EYE camera. (b) Fundus image (video still) from combined D-EYE and Raman spectroscopy setup, highlighting the laser spot, and (c) fundus photograph (video still) focused on the tissue phantom posterior using the combined D-EYE and Raman spectroscopy device. The laser spot in (a) and (b) are indicated by the red arrow. (**B**) Exploded 3D view and schematic (inset) of the eye tissue phantom, consisting of a single lens to mimic the total power of the eye (CM), 4-mm pinhole [iris model (IM)] representing the undilated pupil, and screw in the sample holder (RM). (**C**) Photograph of the 3D-printed tissue phantom (top) and fundus image of tissue phantom using D-EYE camera observing target card at eye posterior (bottom). (**D**) Representative Raman spectra measured from TBI tissue using phantom eye via the portable EyeD setup (top) and from the commercial instrument used to form SOMs and distinguish between control and TBI in the murine model (bottom). The raw data are shown in gray with a smoothed line representation shown in black for visual presentation of the major Raman bands in the high–wave number region. (**E**) Representative OCT images of postmortem [(a) and (b)] and in vivo (**F**) porcine OCT images. Measurement locations at 3000 and 4500 μm are shown as blue and red lines, respectively, illustrating the retinal layers being consistent with a postmortem cellular edema present in (b). Illustrative normal human OCT (c) showing the optic disc margin on the left of the en face image and a horizontal arrow marking the location of the cross section running through the fovea shown on the right. Comparison to the porcine eye reveals the same retinal layers, although the foveal dip is absent in the pig eye.

To measure Raman spectra from the eye posterior segment, a coaligned imaging system was required to target a region of interest on the retina, such as the optic disc. Thus, a D-EYE smartphone fundus camera was used for optical imaging of the retina, which uses the flash from a smartphone camera for illumination and the phone camera for imaging. The D-EYE is a compact optical module providing a direct illumination and therefore can be used without pupil dilation of the patient. A representative fundus image using the D-EYE is shown in Fig. 3A (a). Visual targeting of the laser spot was performed during fundus photography enabling the targeting of a region of interest, such as the optic disc. Figure 3A (b) shows that while a small amount of laser light is transmitted to the camera through the 625-nm filter, during the fundus imaging (Fig. 3A, c), the laser spot is only faintly visible. A crosshair on the phone screen marks the position of the laser, making targeting straightforward and minimizing the laser exposure time (Fig. 3, A and D, inset). A short-pass 625-nm filter, designed for epifluorescence microscopy, is introduced at an angle of 45° into the optical path between the D-EYE camera and the subject. The cutoff range, edge steepness, and efficiency of the filter are such that most of the visible spectrum is transmitted along the path from the camera to the eye and back (>90%) while rejecting white light from the source at wavelengths of >625 nm, which would otherwise interfere with Raman measurements. Wavelengths of >625 nm are efficiently reflected at an angle of 45° (>98%), allowing for the introduction of class I 635-nm laser (0.39 to 0.63 mW) and, subsequently, Stoke’s shifted Raman scatter orthogonal to the fundus imaging path.

The combined system (Fig. 1C) is contained in the 3D-printed housing including the 625-nm short-pass dichroic beam splitter (F3) and the 635-nm laser introduced into the housing via a FiberPort, which provides fine control of the beam position and collimation, aiding alignment. The collimated beam is then passed through a 635-nm laser line filter, before being reflected at 45° by a 635-nm dichroic beam splitter (R2) toward F3 and focused onto the retina by the eye. The backscattered Raman light is reflected along the reverse path of F3 toward R2, where the longer wavelength Raman scatter passes through the filter (R2) to a collection FiberPort (R3). Between R2 and R3, a 650-nm long-pass filter is located to reject Rayleigh scatter to the detector. R3 is used to focus the beam into a fiber, and the spectrum is measured using an Ocean Optics QE Pro spectrometer tailored for a 635-nm excitation. A photograph of the full EyeD prototype is shown in Fig. 1D, demonstrating a compact, portable, and eye-safe system for simultaneous fundus imaging and rapid detection of high–wave number Raman bands.

Our engineered device technology relies on the optical power of the eye to focus the Raman laser (Fig. 1A). To provide a controlled testing environment with fixed optics, a tissue phantom for the eye was further developed to mimic the physical dimensions and optical characteristics of the eye while providing a realistic Raman signature of the retina. On average, the human eye has a combined power of 60 diopters, with the majority of focusing being provided by the cornea and fine adjustment by the crystalline lens (*28*). For simplicity, a single lens is used to mimic the combined power, restricted by a 4-mm diameter pinhole representing the undilated pupil and housed in a 3D-printed case. The tissue characteristics of the retina are then simulated by a removable sample holder, where the tissue can be mounted. The sample holder is screwed in place using 3D-printed threads, which allows for small focus adjustments to compensate for differences in the thickness of different tissue samples. An exploded view and schematic of the lens [condenser model (CM)], housing, and sample/retina model (RM) holder are shown in Fig. 3B, and the combined eye phantom, fundus photography, and Raman spectroscopy optical paths are shown in fig. S3.

The optics of the printed tissue phantom are visually confirmed (Fig. 3C), showing a photograph taken using the smartphone without the D-EYE attachment (top), and with the D-EYE attachment (bottom), where a target card placed at the position of the retina is only visible through the pupil using the D-EYE fundus camera module. Spectra measured from the tissue phantom and optical arrangement from Fig. 1C are shown in Fig. 3D (top), where the major bands of the high–wave number region are resolvable. A representative average spectrum from the training dataset used to identify and cluster TBI in the murine model (Fig. 2, B and C) is shown in Fig. 3D (bottom), where the raw data (gray) were used in the SOM clustering. These results highlight that even while the spectra obtainable from a portable system using a class I laser remain noisy, representative of the real-world conditions at the point of care, data of this quality still provide meaningful insights especially when combined with the use of advanced machine learning algorithm such as SKiNET and a large number of prior training inputs.

**Fig. 4.**
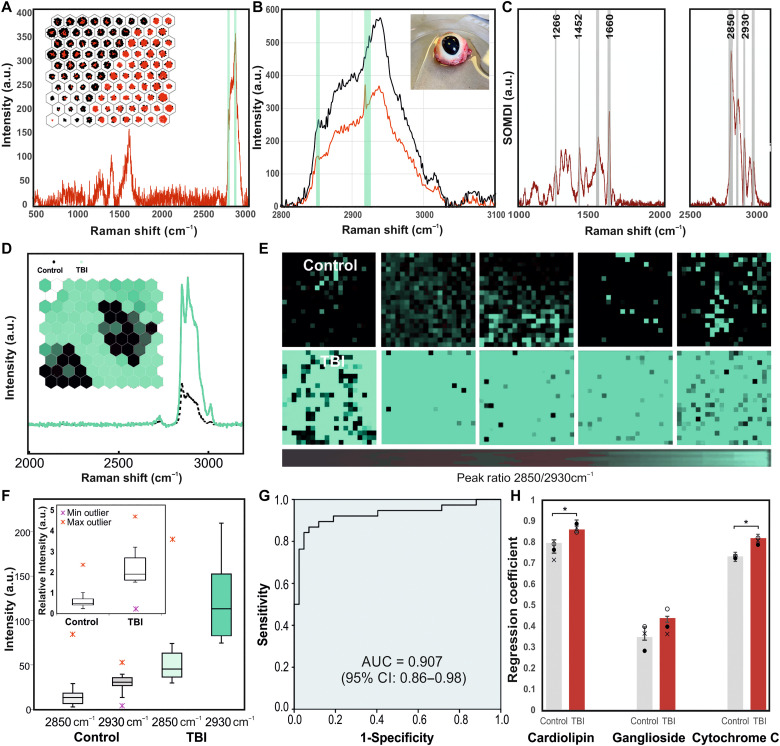
EyeD validation on porcine eye model. (**A**) Representative spectrum of porcine eyes covering the fingerprint and high–wave number regions. (Inset) SOM of the corresponding clustering of Raman spectra from the retina for TBI (red) and controls (black). (**B**) Comparative average spectra from retina of the high–wave number region, collected under the same experimental conditions, using the commercial Renishaw system (black) and the EyeD technology (red). (Inset) Representative ex vivo porcine eye used for Raman analysis, before dissection. (**C**) SOMDI extracted features measured via the Renishaw system [(A), inset] and the EyeD technology [(D), inset], highlighting the most influential peaks for TBI and control groups. Bands highlighted at 1266, 1452, and 1660 cm^−1^ and the ratio of peaks at 2850/2930 are representative of the changes to relative lipid and protein composition as a result of TBI versus control (*80*), generating an overall barcode for the TBI detection via the eye. (**D**) Representative average SOMDI and SOM (inset) of TBI (green) and control (black) of porcine eyes (*n*_Total_ = 51) from retina using EyeD. (**E**) Colored Raman maps of the average peak ratio at 2930/2850 cm^−1^ for eyes from TBI and control groups. Consistent changes observed to spectra in the 2930 cm^−1^ versus 2850 cm^−1^ bands, proportional to injury. (**F**) Box and whisker plots represent the minima, maxima, interquartile ranges, whiskers, and the median in the key-feature peaks of 2930 and 2850 cm^−1^ for TBI and controls and the ratio (inset) of 2930/2850 cm^−1^ levels (*n*_samples_ = 39) versus controls (*n*_samples_ = 12). (**G**) The determined intrinsic classification accuracy from the high–wave number bands of AUC = 90.7 ± 0.9% (inset) (*P* = 0.003), comparable with the receiver operating characteristic curve values detected in biofluids within 0 to 48 hours after injury. (**H**) Changes to lipid composition post-TBI showing the non-negative least squares regression coefficients fitted to the average spectrum collected from each sample (*P < 0.05). The fitting of raw component spectra from brain-specific lipids correlates to SOMDI for a particular state. CI, confidence interval.

The engineered device technology was further used to analyze porcine ex vivo eye retina (Fig. 4B, inset), being closely similar to human eyes in size, structure, development, and composition, enabling the study of the pathological effects of TBI (*39*–*41*).

Before this, a validation study was completed to determine the extent of postmortem structural change in porcine eyes using optical coherence tomography (OCT), a technique commonly used in ophthalmology (Fig. 3, E and F). The postmortem tissues displayed qualitatively less clear separation of retinal layers, with a lower signal-to-noise ratio, which could relate to degradation of the optically clear optical media (such as the cornea) after death as well as to cellular cytotoxic edema occurring in the ischemic and postmortem tissue (Fig. 3E). Objectively, retinal layer thicknesses were higher in the retinal images obtained postmortem (Fig. 3E, b). The relatively small increase (6.4%) in retinal nerve fiber layer thickness and a larger (21.6%) increase in ganglion cell layer thickness (fig. S4A) is consistent with the early cytotoxic edema predominantly affecting the cell bodies with the corresponding SEM thicknesses (fig. S4B). Together these findings are consistent with the abattoir-supplied porcine eyes being in the early stages of postmortem degradation at the time of Raman imaging, with relative preservation of inner retinal structure.

Subsequently, biochemical analyses of porcine retinae were performed. A total of 510 measurements were collected from pigs’ eye retinal samples (*n*_TBI_ = 39, *n*_Control_ = 12). Spectra measured from the retina were taken from an area in close proximity to the optic disc for each eye. Overall, from measurements of the retina, several characteristic bands can be observed in the region of 1200 to 1700 cm^−1^ with an accompanying apparent enhancement of high–wave number bands in the region of 2800 to 3200 cm^−1^, attributed to resonant overtones of vibrational modes in the fingerprint region. Studies have shown that high–wave number region bands can be used to distinguish a number of tissue types, including the difference between myelinated and unmyelinated nerves (*42*–*44*). The resonance effect observed is strong enough to sit above the autofluorescent signal from tissue further supported by being of high enough frequency to be in the tails of the main fluorescence band.

A clear separation between the retina with TBI (red) and control groups can be seen in the SOM shown in the inset of Fig. 4A. Using the SOMDI, it is further possible to identify features in the Raman spectrum responsible for the clustering observed in the SOM. The specific differences in these features originating from biochemical variations in the eye after TBI reflected in the Raman spectra, indicating the changes in molecular composition (Fig. 4, A to C) include the stretching of CH_2_/CH_3_ bonds present in lipids (i.e., cardiolipin, C_81_H_140_Na_2_O_17_P_2_) and proteins (i.e., cytochrome C, C_42_H_52_FeN_8_O_6_S_2_) (CH_2_ symmetric stretching at 2850 cm^−1^ and asymmetric stretching at 2930 cm^−1^; CH_3_ symmetric stretching at 2880 cm^−1^ and asymmetric at 2955 cm^−1^ peaks constituting the gray matter, all identified by SOMDI; Fig. 4C) with the further C═O and C═C coupled bond stretching from the unsaturated fatty acid residues (*45*–*48*). For brain lipids, Raman spectra may be split into regions which originate from the molecular vibrations of different parts of the lipid molecules, i.e., the hydrocarbon tail, the interface region, and the head group (*45*, *49*). The characteristic peaks from TBI spectra compared with the spectroscopic fingerprints of the cardiolipin and cytochrome C (fig. S5) with selected features of six peak intensities and a peak ratio (highlighted as gray lines) form the SKiNET classification, yielding the combined barcode for TBI detection from the retina (Fig. 4C). The peaks detected in the fingerprint region are associated with the changes in the lipid concentration in the brain post-TBI of cardiolipin and cytochrome C, representative of metabolic cellular distress and dysfunction (*48*, *50*–*52*).

Bands originations form the acyl chain of C═O due to the CH_3_ and CH_2_ asymmetric and symmetric stretching vibrations, CH_2_ bending vibrations, the headgroup of ${\mathrm{PO}}_{2}^{-}$ stretching vibration (in the central part of the cardiolipin’s molecular structure) (*45*, *53*), and the interface region due to the C═O stretching vibration can be excellent indicators used to obtain information relating to the conformation of these lipids in the brain. Cardiolipin, playing a key role in cell metabolism and signaling, is known to undergo oxidation during the pathophysiological cascade in TBI, with an accumulation of similar oxidation products in the region of injury as well as comprising the blood-brain barrier, triggering the metabolic disruption and the biochemical cascade following cell damage (*17*, *54*, *55*). Consistent changes are observed in spectra in response to TBI and particularly in the 2850 relative to 2930-cm^−1^ bands. These changes correspond to the C─H and C─C stretching vibrations as the trans-gauche in the hydrocarbon is altered and as structural changes occur in the hydrated lipids, and the intensity of bands near 2930 and 2850 cm^−1^ alters due to asymmetric and symmetric stretching vibrations, respectively. Such hydrocarbon chain transitions are accompanied by discontinuous changes in both wave number of the bands and the bandwidths, where the absorption maxima and bandwidth increase indicating greater hydrocarbon chain disorder and the start of the change to the gauche form. In this form, the band at 2850 cm^−1^ is weakened due to the vibrational decoupling. Further peaks identified as strong SOMDI weights are associated with bands at 1452 and 1660 cm^−1^ from to the scissoring and wagging vibrations of CH_2_/CH_3_ and the ${\mathrm{PO}}_{2}^{-}$, indicative of the changes occurring in the cardiolipin following a TBI. These bands are sharp when the lipids are in the trans-configuration and become broad as the conformational changes proceed with the overall intensity decreasing. The frequency of the P═O group (within the O═${\mathrm{OP}}_{3}^{-}$ of cardiolipin) is further influenced by the number of electronegative substituents directly bonded to it as well as being sensitive to association effects (*45*) and therefore results in a shift in band position of about 40 cm^−1^, further enhancing the 1660-cm^−1^ peak (*45*, *51*, *52*).

Furthermore, the strong Raman intensity of the peak at 1660 cm^−1^ in Fig. 4 (A and C) corresponds to C═N stretching vibrations of cytochrome’s C pyrroline (*56*). There is also a very weak band of the *S*─H stretching vibration in a region relatively free of absorption bands at 2600 cm^−1^ associated with the mercaptan of the cytochrome C. This protein is known as a biomarker indicative of cell death apoptosis and is found in the mitochondrial inner membrane, where a complementary change to cardiolipin is expected. Membranes of cells are the primary target for injury and their damage and are highly dependent on their physical properties and lipid organization, affecting membrane fluidity, which is a key property for maintaining cell functionality and depends on lipid composition and cell environment, leading to distortions, deformations, and decrease of mechanical stability (*57*–*59*). Cardiolipin undergoes oxidation during the pathophysiological cascade in TBI, with an accumulation of similar oxidation products in the region of injury (*38*, *60*). A further link has been established between the spectral changes and apoptosis via comparison to immunohistochemistry of TBI in mice using Raman spectroscopy (*33*). An accumulation of ganglioside in the region of injury has also been demonstrated (*57*).

Given the brain’s high-fat content, with the Raman signatures for the major and minor brain-specific lipids being well-characterized (*61*, *62*), we applied the non-negative least square fitting versus the average spectra from the retina (Fig. 4H) and the raw data of brain-specific lipids in the range of 1200 to 3000 cm^−1^, to identify the relative contributions in each sample for TBI and control groups. The resultant fitting coefficients for each spectrum are proportional to the biomarker concentration measured within each retina sample. From the decomposition of contribution from brain lipids in average Raman spectra of retinal samples, four main lipids including cardiolipin, ganglioside, cytochrome C, and cholesterol, have exhibited coefficients with a value above zero. Among these, the most statistically significant difference has been identified from the contribution of cardiolipin, linked to the increase in the peaks’ ratio of 2930/2850 (Fig. 4, B to D and F) in TBI versus the control. As TBI occurs clinically, the lipid and protein contents in the eye increase, and the peaks originating from these become more pronounced in the Raman spectra. The ratio was elevated in the eyes after TBI [median, 2.34; interquartile range (IQR), 0.63; *P* < 0.0010] compared with the control group (median, 0.48; IQR, 0.12). The central line in the box plots (Fig. 4F) represents the median, top and bottom edges of the box are the upper and lower quartiles, whiskers extend to upper and lower quartiles plus and minus 1.5× the interquartile range, and crosses indicate values outlying the whiskers. A further statistically significant change also is evident in cytochrome C post-TBI compared to the control. There is no statistically significant difference between TBI and control groups for ganglioside, and only a small decrease in the fitting coefficient observed for cholesterol, most probably a result of a hemorrhage, yielding an increase in its concentration specific to the injury site; however, it was not statistically significant.

Two closely related aspects can be derived from the above Raman analysis. First, the spectral changes related to hydrocarbon chain conformation and packing, forming the base for the above peak assignments and the corresponding discussion, and second, spectral changes related to gross changes in the environment of the hydrocarbon chains. It has been previously shown that there is an increase in cerebral cortical free fatty acids (used as further predictive markers of early outcome) following cortical impact brain injuries in rats (*63*) and human cerebrospinal fluid (*64*), suggesting that these phospholipases, activated by the TBI, hydrolyzed several phospholipids within minutes of the injury. Changes in the environment of hydrocarbon chains of lipid molecules result in strong effects on the C─H stretching vibration region of the Raman spectra, and these can be further used to indicate different states of order, making it possible to detect whether the hydrocarbon chains of the lipid molecules are associated into separate lipid regions or located in a protein environment. When the hydrocarbon chains of the lipid cardiolipin are in a natural state, the symmetric stretching vibrations of the CH_2_ groups at the 2850-cm^−1^ band dominate. However, when the cardiolipin undergoes environmental change, such as in the case of TBI, the relative intensity of the 2930-cm^−1^ band, in relation to the other C─H stretching vibration, is increased relative to the intensity of the 2850 cm^−1^ as well as compared to the 2885-m^−1^ peaks in hydrocarbon chain region (*65*), due to the disorder induced in the hydrocarbon chains. As the environment changes increase and becomes more polar during the pathophysiological cascade in TBI, the 2930-cm^−1^ peak increases successively compared to the other C─H stretching vibration peaks. This is associated with the increased importance of the intermolecular interactions for the C─H stretching vibrations than the coupling along the carbon skeleton of the chain, which affects the C─C stretching vibrations. Hence, a small change in the lateral packing of largely disordered hydrocarbon chains, i.e., from being freely dispersed in healthy controls in a plane to being forced to be reorganized into a more spherical micelle-like state of order due to the metabolic cascade following TBI. The increase in the cardiolipin concentration, as well as the presence of the cytochrome C, provides sufficient change in the neighborhood of the CH_2_ and CH_3_, yielding detectable changes in the 2930/2850 peak ratio.

The feature bands in the barcode, identified as strong SOMDI weights as derived from the analysis provided by SKiNET, closely reflect the cardiolipin and cytochrome C molecular structures and the corresponding variations, indicating that, in the earliest stages after TBI, the concentration ratio of these biomarkers’ changes in the retina, providing a prediagnostic value to the in-hospital histopathological outcomes.

To assess the ability of the EyeD to differentiate TBI via the retinal changes, the area under the curve (AUC) for each peak and their ratio was calculated (Fig. 4G, inset) with the true-positive rate against the false-negative rates was plotted. From the SKiNET model optimization with the 10-fold cross-validation on the training data, the determined intrinsic classification accuracy for the peak 2930/2850 ratio was found to be AUC = 90.7 ± 0.9%, with normal based, two-sided 95% confidence interval of less than ±9%, clearly discriminating between TBI and control groups, comparable to the receiver operating characteristic curve of the sensitivity versus 1-specificity derived following TBI detected from biofluids within 0 to 48 hours (*16*). The AUC indicates that the change in the peak ratio of 2930/2850 following the TBI could be a valuable indicator for discriminating TBI from healthy control cohorts.

**Fig. 5.**
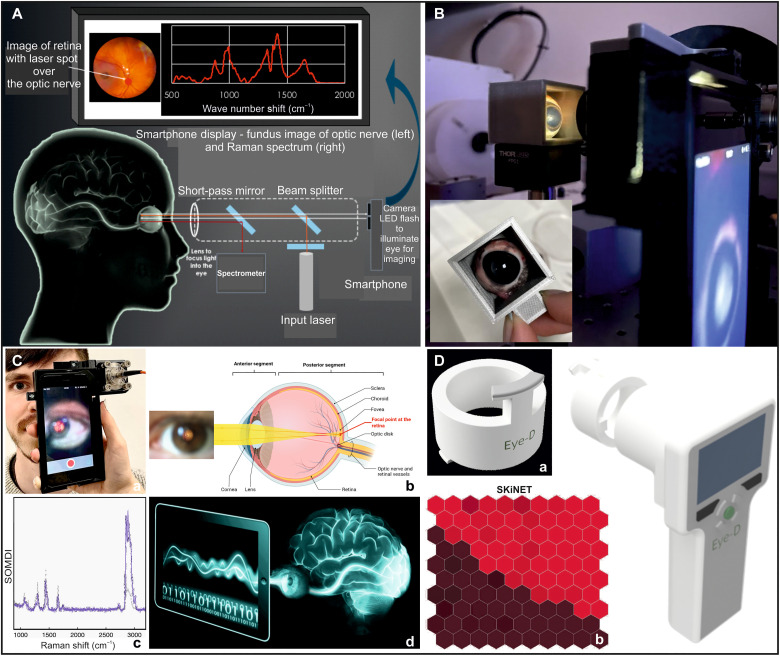
Translatable neuro-engineered EyeD technology for rapid TBI point-of-care diagnostics. From (**A**) concept through to the (**B**) design of the EyeD with a 3D-printed phantom eye-model (with the synthetic eye model incorporated into the housing design) mimicking the optical properties of the eye, allowing Raman spectra through an eye-like lens to be acquired on model samples and optimizing of the resolution and optical throughput and onto the (**C**) lab prototype, which uses the eye to focus a collimated beam onto the retina, enabling measuring the spectroscopic fingerprint bands while concurrently performing fundus imaging to verify that the correct area is imaged [(a) and (b)] created using BioRender. Subsequently, SKiNET generates (c) feature extraction and identification of the most important Raman bands by separating the high-dimensionality data from different classes, identifying the underlying chemical differences, and classifying the data using the network with peaks in SOMDI corresponding to cm^−1^ and modes that contribute most to the clustering observed in SOM. Its implementation using open-source libraries enables data analysis to be performed online and run from any browser or smartphone (d), reducing the hardware footprint and increasing portability. (**D**) The engineered EyeD incorporates a (a) disposable eye guard, (b) SKiNET software for rapid data classification, and visually clear on-site readout for clinicians or paramedics, enabling automated interpretation of Raman data and thus markedly improving the speed and cost of diagnosis at the point of care using an (c) ergonomic handheld device for simultaneous fundus photography and high–wave number Raman spectroscopy.

In summary, we have demonstrated an unprecedented concept of measuring changes to brain neurochemistry noninvasively via the eye, overcoming the strict constraints of in vivo imaging that are highly unfavorable for Raman spectroscopy, and shown the first evidence that spectra of the neuroretina can be used to identify TBI. We show that by using the eye alone to focus a collimated beam onto the retina, high–wave number Raman bands can be measured while simultaneously performing fundus imaging (Fig. 5). The engineered eye phantom, mimicking the physical dimensions and optical characteristics of the eye, further opens a new avenue for further straightforward studies of various biological tissues in conjunction with Raman spectroscopy, enabling validation of developments for other detrimental neurological and ophthalmological diseases.

We have established that high–wave number bands alone can be used to identify TBI from the retina and subsequently designed a portable device for eye-safe data acquisition in a realistic synthetic model of the human eye, providing the first tangible path toward noninvasive point-of-care diagnostics of the brain using Raman spectroscopy. The engineered EyeD technology combined with SKiNET has been then used to investigate whether the retina can reflect the brain microenvironment after injury, in clinically relevant murine and porcine eye models of focal TBI. The intensity ratio of two main peaks at 2850/2930 cm^−1^ was found to be associated with the CH_2_ stretching of lipids/CH_3_ symmetric stretching of proteins, further revealing information on the type of lipid-protein biomarker interaction due to the metabolic cascade following TBI, the environment of the hydrocarbon chains of the lipid, and the state of order of the chains. Retinal degeneration after TBI is directly caused by CNS damage affecting the visual pathways, accounting for 30% of the cerebral cortex (*66*–*68*). The detected retinal changes are found to closely associate with TBI, eliciting subtle spectral changes through the use of multivariate analysis, linked to variation in cardiolipin and cytochrome C, and indicating metabolic disruption. These lipid-rich fatty substances encompass the brain tissue (*69*), especially with the brain containing nearly 60% fat and Raman signatures for all 12 major and minor brain-specific lipids being well characterized (*61*, *70*). The characteristic peaks from the porcine eye with selected features of the 2850/2930 ratio and six peak intensities in the fingerprint region form the multilayer classification yielding the combined spectroscopic barcode for TBI detection via the EyeD technology. While Raman data show a strong and uniform relative change for severe TBI, tissue from the mild TBI retina appears more heterogeneous. From pigs’ eyes, more obvious changes were observed to spectra in response to injury, which is in contrast to the more subtle changes seen in the murine model. The consistency in the findings between large and small animal models, with different injury mechanisms and different tissue processing methods, strongly supports the veracity of these findings. The greater magnitude of changes in the porcine model may further relate to the unfixed nature of the tissue as well as to the model involving a more severe TBI.

In the handheld technology, integrated SKiNET can simultaneously provide dimensionality reduction, feature extraction, and multiclass classification to act as a decision support tool to allow automated interpretation of Raman data without specialist support, markedly improving the speed and cost of diagnosis (Fig. 5). The achieved SKiNET classification performance confirmed via cross-validation results from SOM, and the corresponding discriminant indices are therefore a direct reinforcement of the SOMDI observations, ensuring the high reproducibility. Smartphone camera–based systems are easy to use and, coupled with AI diagnostic support, produce an output when an adequate signal is obtained. The experience of using smartphones to acquire images is ubiquitous and will lead to a high user acceptance. Clinical efficacy studies will determine the extent to which paramedics and clinicians rely on the device to drive decision-making. The EyeD readout would form part of a protocolized decision-making tree such as: normal EyeD + no “red flag” symptoms or signs classify the head injury as mild TBI not requiring hospital assessment versus abnormal EyeD mandates additional assessment in emergency department. Further, by levering modern web technologies, data analysis can be performed remotely on any device that has a web browser and is designed to be user friendly to clinicians (*61*).

In comparison to the currently used methods in ophthalmology for TBI diagnostics, there are no comparable techniques in clinical practice. OCT and fundus photography as well as B scan ultrasound provide structural imaging of the retina. Raman spectroscopy provides information on the biochemical composition and therefore the metabolic state of the neuroretina, which is only indirectly available with structural imaging modalities, such as using OCT angiography or fluorescein angiography, which may reveal reduced blood flow, from which clinicians could infer retinal ischemia. While magnetic resonance spectroscopy is unable to examine the retina due to the minimum voxel size being too large, it could examine the vitreous and has occasionally been used for this purpose in research; however, there is a limited relationship between vitreous and retinal findings. A brief overview of ophthalmological technologies for the assessment of TBI is summarized in text S1.

To date, the specificity in multidisease settings has not been sufficiently established. All approaches used clinically today and many of the proposed methods, e.g., S100B, lack specificity (*30*), and while the latter has been implemented in Scandinavian countries, it is rarely used elsewhere (*71*). The high sensitivity for the combination of GFAP and neuro–Ubiquitin C-Terminal Hydrolase L1 (UCH-L1) measured within 12 hours of injury (*72*), formed the basis of the first US Food and Drug Administration–approved TBI test for triaging the need for CT, however, is affected by poor specificity (36.5%), low-positive predictive value (9.2%), and long-analysis times, defeating its purpose (*73*). GCS, despite being highly subjective, remains the only ground truth for clinical and academic understanding of TBI that can span the entire patient journey as well as injury severity. Measuring the biochemical compounds via EyeD from the retina directly, as an accessible part of the CNS, circumvents many of the drawbacks of TBI detection, particularly with specificity. Known biomarkers once thought to be highly specific for the brain have later been found to have additional extracranial sources, which confound the results. This a particular issue in polytrauma. In the case of the compounds detected by the EyeD, there is no biological reason to infer that extracranially derived metabolites would accumulate in any large quantities in the retina, as the blood-retinal barrier prevents the entrance of systemic compounds from circulation. We thus measure the changes directly from the blood-brain-barrier side, probing both the local brain biology via the neuroretina and the global brain via the optic nerve. Thus, our noninvasive in vivo spectroscopic EyeD technology, which could be combined with a parallel blood test (*16*), would be reflective of both global brain pathology (via probing the optic nerve) and any local neurological disease (via detecting from the neuroretina), representing the first opportunity for an alternative to GCS while simultaneously offering greater fundamental mechanistic insights to further our understanding of the underlying pathobiology of TBI.

Furthermore, the potential scope of in vivo EyeD detection to characterize the neuroretinal sequelae of trauma is wide. Retinal degeneration after TBI is directly caused by CNS damage involving the visual pathways, which comprise 30% of the cerebral cortex (*74*, *75*). While the retinal changes detected in animal models closely associate with TBI severity, the markers considered (and other potential markers) detected in the retina may also associate with local pathology and afferent visual dysfunction. Given that up to 80% of patients with mild TBI have long-term visual complaints, the ability to detect and predict long-term visual dysfunction after TBI would further be extremely valuable. It is also possible that the detected metabolites may reflect more generically an acute brain injury. This would not affect the utility of the solution in the context of trauma, as the test would not be used in a total vacuum of information. The identification of a generic acute brain injury would still call for neuroprotective measures and triage to a neuroscience center, whatever the cause, and could theoretically be of use in many acute neurological conditions (e.g., stroke or the “found unconscious” patient). Ocular Raman spectroscopy can be further translated to other clinical applications in ophthalmology and neurology, such as the early diagnosis of diabetic retinopathy and dementias or monitoring drug delivery to the brain.

In the longer term, the EyeD technology has the potential to offer crucial clinical insight in a growing number of diagnostic and patient-monitoring scenarios. By developing an eye-safe fundamental mechanism that combines fundus imaging and Raman spectroscopy to allow reliable retinal data acquisition, we bridge the translational gap via additive manufacturing, smartphone technology, and machine learning. Our results highlight that Raman spectroscopy of the neuroretina is subject to the natural optics and dimensions of the eye but show how this can be incorporated into the device design. While we have demonstrated that high–wave number bands detected in the eye can be used to diagnose TBI, Raman spectroscopy EyeD has the potential to also be applied to a multitude of neurological conditions. The measurements are made portable and noninvasive, therefore enabling routine point-of-care use and long-term patient monitoring. As a pathway to translation, the standalone spectrometer will be replaced with a compact on-device spectrometer and smartphone readout, allowing for fundus photography and Raman spectroscopy from a single smartphone screen, backed by cloud data processing, storage, and machine learning. The final portable EyeD will be used to detect neurotrauma at point of care, e.g., roadside, pitch side, and austere combat environment, where no expert evaluation or urgent radiological investigations are immediately available. This has the potential to revolutionize how TBI and neurological conditions are diagnosed and triaged, which, in turn, would provide substantial health care savings and improved clinical outcomes and save many lives.

## MATERIALS AND METHODS

### Porcine eye study

Pig eyes were acquired from large white pigs (Liverpool Medical Meat Supplies). Traumatic focal brain injuries, representative of a cortical impact, to the pigs’ brain (frontal and parietal) were induced as an electrical stun shock directly to the brain, followed by an incision to the neck [following United Kingdom Food Standards Agency (UK FSA) health guidelines as part of standard abattoir slaughtering practice]. These injury forces led to clinically relevant histopathological outcomes including cellular damage and death, skull fracture, disruption and hemorrhage of the cortical surface, swelling, edema, and contusions (*76*), representing the head injuries acquired from falls, which account for over 35% of all sustained TBIs and over 50% of TBIs in children younger than 14 years (*77*). Control eye samples were acquired from Yorkshire pigs dying a natural death. All eyes remained optically clear (Fig. 4B, inset) and were analyzed 2 to 4 hours after extraction. Retinae were dissected out and analyzed in flat mount on aluminum-covered microscope slides. In determining the extent of changes in the retinal structure of living and postmortem eyes of Yucatan and Yorkshire pigs, respectively, eyes were imaged using a Heidelberg Spectralis OCT platform (for both postmortem and in vivo imaging), using the manufacturer’s “Posterior Pole” protocol to acquire volumetric retinal scans in 61 horizontal b-scans averaged over at least nine frames in a 30° × 25° volume centered temporal to the optic disc in the expected location of the pig visual streak. OCT images were exported in tiff format and analyzed using ImageJ. The retinal nerve fiber layer and ganglion cell layer were manually segmented, and thicknesses were measured at 3000 and 4500 μm from the optic disc orientated in the axis of the visual streak. Raman spectra were acquired using both the InVia Qontor (Renishaw Plc) and the engineered EyeD technology equipped with a 635-nm laser. Surface maps over an area of 2500 μm^2^ were acquired for each sample, with an acquisition time of 3 s and using a 50× Leica objective (0.75 NA), with scans recorded in the ranges of 500 to 3500 cm^−1^ and 2032 to 3466 cm^−1^, accordingly. A total of 400 spectra per tissue sample were recorded. Spectra were processed using cosmic ray removal and baseline subtraction in WiRE 5.3 (Renishaw Plc) and exported to text files.

### Murine samples

Murine samples were purchased from the Istituto di Ricerche Farmacologiche Mario Negri IRCCS following experimental brain injury and spectra measured from flat mounted retina samples, as we have previously described (*17*, *29*). Briefly, the brain injury was induced using a 3-mm rigid impactor driven by a pneumatic piston rigidly mounted at an angle of 20° from the vertical plane and applied to the exposed dura mater, between bregma and lambda, over the left parietotemporal cortex (antero-posteriority: −2.5 mm, laterality: −2.mm), at an impactor velocity of 5 m/s. The deformation depth was of either 1 or 0.5 mm, resulting in a severe or moderate level of injury respectively. The craniotomy was then covered via cranioplasty, and the scalp was sutured. Sham mice received identical anesthesia and surgery without brain injury. Three days after TBI, mice were deeply anesthetized with ketamine chlorhydrate (20 mg, i.p.) and medetomidine chlorhydrate (0.2 mg, i.p.) transcardially perfused with 30 ml of 1% phosphate-buffered saline (PBS) (pH 7.4), followed by 60 ml of 4% paraformaldehyde (PFA) in PBS. The brains and eyes were carefully removed from the skull and postfixed in 4% PFA in PBS for 24 hours at 4°C. The postfixed tissue was then rinsed and stored in normal saline (NaCl 0.9%) at 4°C. Adult (8 weeks old) C57BL/6J male mice (Envigo RMS Srl) were used. No additional procedures were performed on mice except those related to the experiment they were intended for. Procedures involving animals and their care were conducted in conformity with the institutional guidelines of the Istituto di Ricerche Farmacologiche Mario Negri IRCCS, Italy in compliance with national (D.lgs 26/2014; authorization no. 19/2008­A issued by Ministry of Health) and international laws and policies (EEC Council Directive 2010/63/UE; the National Institutes of Health Guide for the Care and Use of Laboratory Animals, 2011 edition). They were approved by the Mario Negri Institute Animal Care and Use Committee that includes ad hoc members for ethical issues and by the Italian Ministry of Health (Decreto no. D/07/2013­B and 301/2017­PR). Animal facilities meet international standards and are regularly checked by a certified veterinarian who is responsible for health monitoring, animal welfare supervision, experimental protocols, and review of procedures.

### Artificial neural network data analysis and classification

SKiNET, an open-source analysis tool (*25*) with an accompanying Raman Toolkit web interface, was used to generate SOM models from the training data and perform predictions against the test data. A total of 20% of the data was randomly selected from each group and used as test data with the remaining 80% of data used for training (tables S1 and S2). To achieve higher accuracy of the SOM size, these models were optimized by performing cross-validation on the training data, tuning the number of neurons (hexagons), initial learning rate, empiric testing of the number of epochs, and number of training steps, with classification accuracy determined using a 10-fold cross-validation. To determine the best matching unit, the initial area size was maintained at two-thirds the edge length of the grid with the cosine similarity used as the distance metric. The 400 spectra measured across each sample were grouped according to class (table S1). A total of 20% of the data was randomly selected from each group and reserved as test data, leaving the remaining 80% for training (table S2). Analysis of the training data was performed using SKiNET by randomly passing samples from the training data into the SOM over a number of iterations. SKiNET models were optimized by performing 10-fold cross-validation on the training data and tuning the number of neurons, initial learning rate, and number of training steps. The final model used a 10 by 10 or a 20 by 20 grids of neurons (for each group), 57,600 training steps (nine epochs), with an initial learning rate of 0.3. The initial neighborhood size was maintained at two-thirds the edge length of the grid, and cosine similarity was used as the distance metric to determine the best matching unit. Last, the optimized model was used to classify the previously unused test data, to give an indicator of the classification performance. Classification using the test data were repeated 10 times from separate SOM initializations and an average of the results output as a confusion matrix. A repeat initialization of the classification verified the stability of the model.

### Non-negative least squares analysis

Non-negative least squares analysis was performed on eye samples by fitting a library of component spectra to the average spectrum for each sample (table S3). The component spectra consisted of raw data for brain lipids and cardiolipin (*70*). Cytochrome C was purchased from Sigma-Aldrich Ltd. and measured without modification at 633 nm using a laser power up to 1 mW, focused through a 50× Leica objective (0.75 NA) over 3 s (1-s acquisition, 3 accumulations). The *lsqnonneg* function in MATLAB was used to determine coefficients of the raw component spectra to the average spectra measured from the eyes. The *interp1* function was used to rescale the data in increments of one inverse centimeter. Raw component spectra from brain-specific lipids were fitted to SOMDI for a particular state, constituting a physically realistic fit, as Raman spectra represent a mixed state of positive contributions from constituent components. The change in fitting coefficients was used to interpret the compositional changes to the retina in response to the injury.

### Raman spectroscopy

InVia Qontor confocal Raman (Renishaw) spectrometer equipped with 514-, 633-, and 785-nm lasers, which was adjusted for optimal throughput, fluorescence control, and sensitivity, was used to acquire the standard and comparative data. Normalization was applied so that the AUC of the spectrum equates to 1 in each case and the data to be plotted on the same scale, enabling a straightforward comparison between spectra taken from instruments with different optics. The acquired spectra were normalized using the standard normal variate, and cosmic ray peaks were removed using a custom Python script (Python 3.7) and WiRE 5.3 (Renishaw Plc). Raman maps were generated in a Streamlinemode scan with 1-s acquisition, 3 accumulations at 633 nm. A 50× objective with an NA of 0.75 was used for Raman measurements. Optical measurements were carried out with a specially adapted research grade microscope (Leica DM 2700 M) equipped with an incoherent white light source, allowing confocal measurements with 2.5-μm depth resolution. Postprocessing of spectra was performed in WiRE 5.3 and Python 3.7, and cosmic rays were removed from each map using the nearest neighbor method, followed by baseline subtraction using the “intelligent spline” fitting (11 nodes). The average was taken from each map resulting in a single spectrum per sample.

### Design and fabrication of the EyeD technology

Computer-aided design (CAD) designs for 3D printing were made using Autodesk Fusion 360 (figs. S2 and S3) and printed in polylactic acid using an Ultimaker 3 Extended (Ultimaker BV).The combined Raman spectroscopy/fundus photography setup consisted of the following components: iPhone (Apple Inc.), D-EYE Smartphone-Based Retinal Imaging System (D-EYE Srl), 625-nm edge BrightLine single-edge short-pass standard epi-fluorescence dichroic beam splitter, 635-nm BrightLine dichroic beamplitter (Laser 2000 Ltd.), 650-nm FEL0650 long-pass filter, 635-nm FL0635-10 laser line filter, 2× FiberPort, FC/PC 100-μm 0.22-NA multimode input fiber, SMASMA 100-μm 0.22-NA multimode output fiber (ThorLabs Inc.), 635-nm class I laser (KI9807A VFL, Kingfisher International) (fig. S6) (*78*), and QE Pro Spectrometer optimized for 638 nm (Ocean Optics Inc.). The eye tissue phantom consisted of a 3D-printed housing encasing an aspheric condenser lens (ACL2018U) with a focal length of 18 mm (ThorLabs Inc.). Fatty tissue from bacon was used to simulate the signal from the optic nerve and retina in the phantom. Fundus photograph of a human eyes (approved by University of Birmingham ethics committee for healthy controls, Ethics Reference: ERN_22-1129) were taken using unmodified D-EYE camera attachment. Further (OCT/fundus) images were from patient recruited to the Ophthalmic and Neurocognitive Assessment in the Management of Critically Ill Patients study: 19/YH/0113, approved by the National Health Service Research Ethics Service and conducted in the Ophthalmology Department at the Queen Elizabeth Hospital Birmingham of University Hospitals Birmingham NHS Foundation Trust (UK), as previously described. Inclusion criteria were patients over the age of 18 years with planned esophagectomy. Exclusion criteria were individuals with preexisting retinal pathology, optic nerve pathology, or known neurological conditions. Patients were approached in clinic by members of the clinical care team, and if they expressed a willingness to participate, were given a patient information leaflet, had the opportunity to discuss the study, and were invited to participate by a member of the research team, providing written, informed consent if they agreed. All documents were approved by the NHS Research Ethics Service and the Hospital Trust. Spectra were acquired using OceanView software (Ocean Optics Inc.) and an acquisition time of 30 s and 3 accumulations. An intelligent-fitting filter was applied for baseline subtraction. After excluding regions with peaks, the baseline was fitted to all the remaining points in each spectrum, and a polynomial order of nine with the noise tolerance of 1.50 was applied. The baseline correction was achieved via the modified polynomial fitting (ninth order) and optimized peak detection, as auto baseline default configuration was not suitable, providing a close-fitting correction and background subtraction.

### Receiver operating characteristic curves and box plots

Receiver operating characteristic curves were generated from the acquired data for different cutoff points using nonparametric Mann-Whitney *U* and Kruskal-Wallis tests using SPSS (*79*). Each point in the receiver operating characteristic curve represented a sensitivity-specificity pair corresponding to a particular decision threshold, and the values of sensitivity, specificity, and accuracy were calculated using standard equations. A test with perfect discrimination (no overlap in the two distributions) exhibited a receiver operating characteristic curve that passed through the upper left corner (100% sensitivity and 100% specificity) and the closer the receiver operating characteristic curve was to the upper left corner, the higher was the overall accuracy of the test. Box plots were generated using Vertex42 software. Each series was an *x-y* chart used to represent the quartiles, allowing the data to include negative values. The median was denoted with the “*x*” marker, and horizontal markers were used for quartile 1 and quartile 3 without requiring shifting of the data. The comparison of the TBI group with the control group was performed using a two-sided normal-based 95% confidence interval *t* test, and *P* < 0.05 was considered significant. Classification sensitivity, accuracy, and specificity were determined on the basis of detection results: sensitivity = (TP)/(TP + FN), specificity = (TN)/(TN + FP), and accuracy = (TP + TN)/(TP + TN + FN + FP) where TP is “true positive,” TN is “true negative,” FP is “false positive,” and FN is “false negative.”
