# Supplementary material for: Window into the mind: Advanced handheld spectroscopic eye-safe technology for point-of-care neurodiagnostic
Source: Sci Adv. 2023 Nov 15;9(46):eadg5431. doi: 10.1126/sciadv.adg5431 (PMC10651125; doi:10.1126/sciadv.adg5431)
Supplement: Supplementary file 1 — Figs. S1 to S6 Tables S1 to S3 Supplementary Text S1 [file sciadv.adg5431_sm.pdf]

Science Advances
AAAS

Supplementary Materials for

Window into the mind: Advanced handheld spectroscopic eye-safe technology for point-of-care neurodiagnostic

Carl Banbury *et al.*

Corresponding author: Pola Goldberg Oppenheimer, goldberp@bham.ac.uk

Sci. Adv. **9**, eadg5431 (2023)
DOI: 10.1126/sciadv.adg5431

This PDF file includes:

Figs. S1 to S6
Tables S1 to S3
Supplementary Text S1

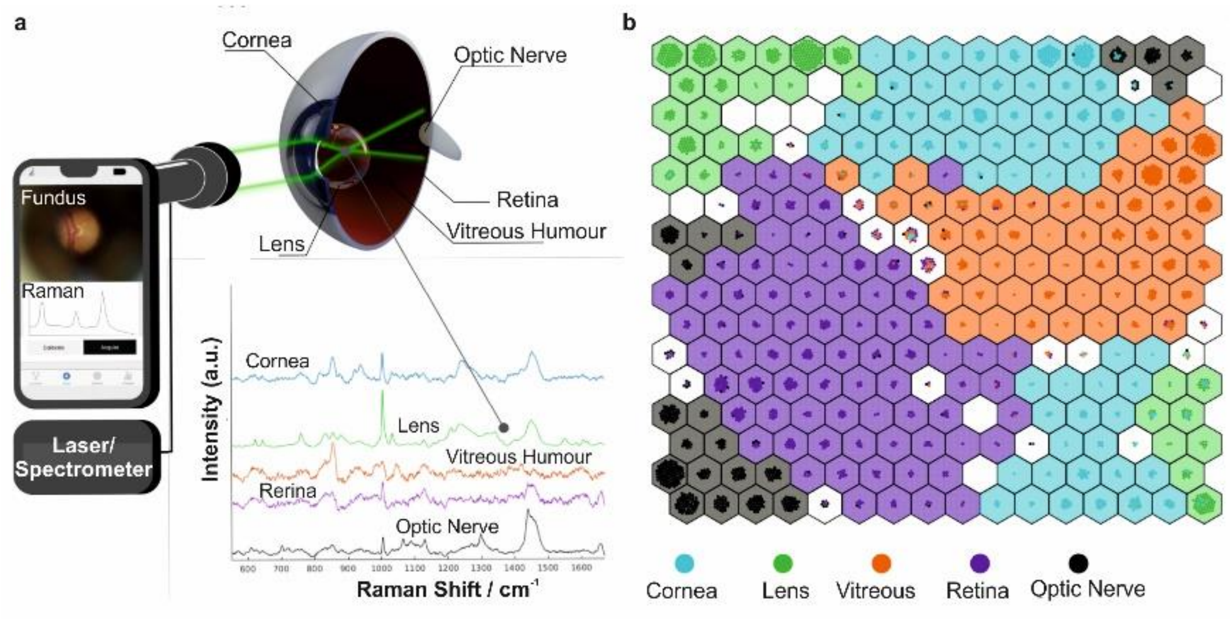

Supplementary Figure S1. Fundus imaging the eye, coupled to Raman spectrometer and connected to cloud-based artificial neural networks for analysis and SOM classification from anatomical layers in the eye by SKiNET. (a). Fundus imaging the eye, coupled to Raman spectrometer and connected to cloud-based artificial neural networks for analysis with representative spectra from anatomic eye segments illustrating how the combined SkiNET is applied to auto-detect the plane of focus from a Raman measurement of the eye, using the information to reject signal from unwanted eye layers. **(b).** The SOM defines a 2D map of neurons, typically arranged as a grid of hexagons. Each neuron is assigned a weight vector, which is initialised randomly and has a length equal to the number of variables in a spectrum. The weight vector affects which neuron will be activated for a given sample and neighbouring neurons will have similar weights. Spatial clustering is therefore observed in the trained map, with spectra that exhibit distinct properties activating different neurons. The developed method is based on the self-organising maps discriminant index (SOMDI) that performs visual separation of N class types to identify the underlying chemical differences between classes and classifies data. The peaks in SOMDI correspond to Raman vibrational modes that contribute most to clustering.

Supplementary Table S1. Summary of spectra used as inputs for multivariate analysis across the studied groups.

Group	Spectra per Sample	Samples	Total
**TBI**	10	39	390
**Controls**	30	12	360
**Total**			**750**

Supplementary Table S2. Breakdown of data across each class and split into training and test data sets. Classification using SKiNET algorithm, providing classification accuracy of 90.1%.

Group	Total	Training Data	Test Data
**TBI**	390	78	312
**Controls**	360	72	288
**Total**	**750**	**150**	**600**

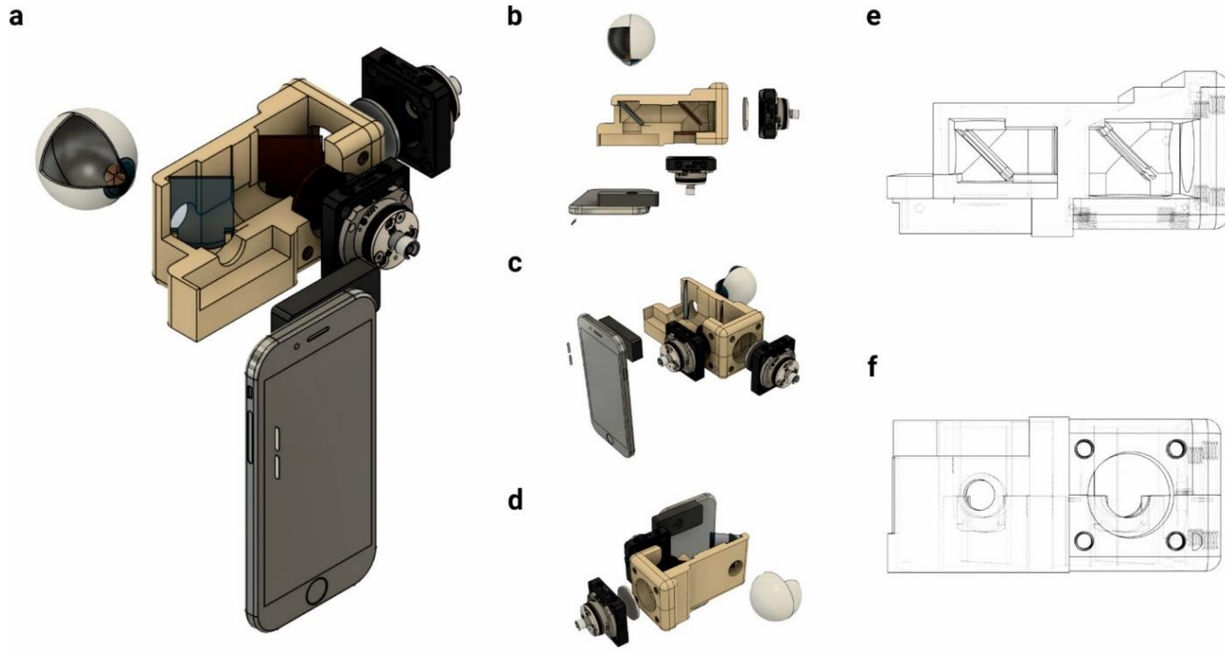

Supplementary Figure S2. Three-dimensional Designs of the EyeD. Schematics of the 3D printing design for housing a portable Raman system with a possibility of incorporating the eye model. LEGO style pins, visible in each corner help align and lock a lid section in place. **(a-d)** EyeD housing with accessories in shaded with visible edges only visual style **(a).** Perspective view with all accessories fully or partially visible. Slot for the D-EYE attachment and a clear path directly to the eye sample. **(b).** Top view of the alignment of each component with both dichroic beam splitters set at 45° to the beam path. **(c).** Side view of both fibre ports with clean-up and Rayleigh filters mount the housing **(d).** Back view illustrating the unobstructed beam path out of the system into the eye, with no components touching the patient's face. **(e-f).** EyeD housing without accessories in wireframe with hidden edges visual style **(e).** Top view of the open top sections to allow accessories to be adjusted, replaced or cleaned, along with the raised slots that secure the dichroic beam splitters into place. **(f).** Side view illustrating **(left)** the entrance aperture of the D-Eye camera all the way through to the exit at the eye location and **(right)** the input fibre port mount where the laser enters the housing.

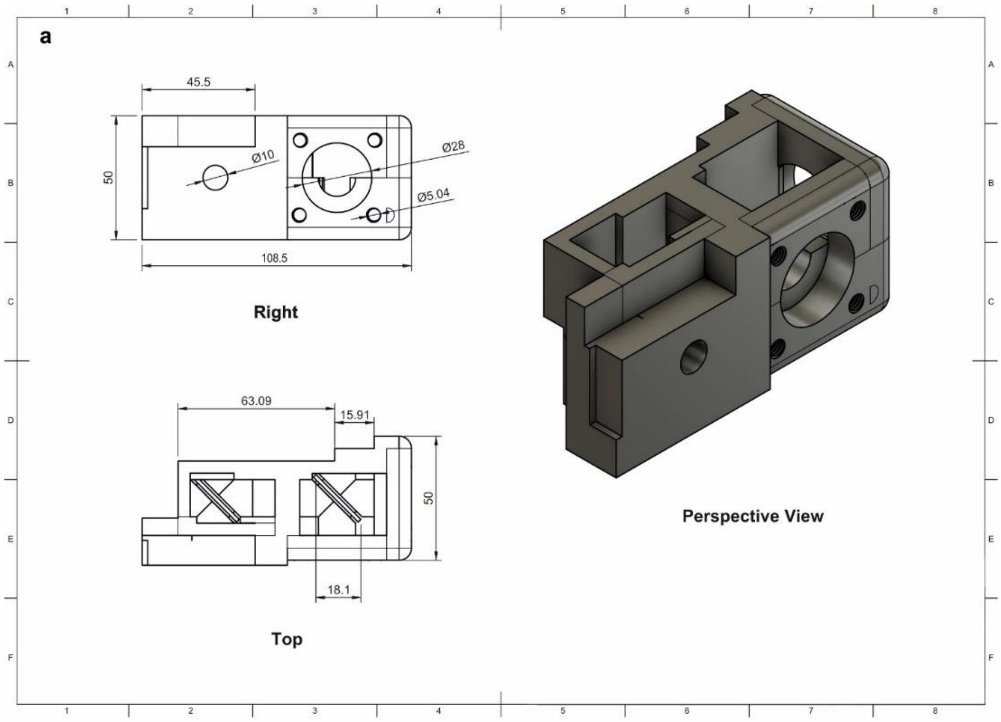

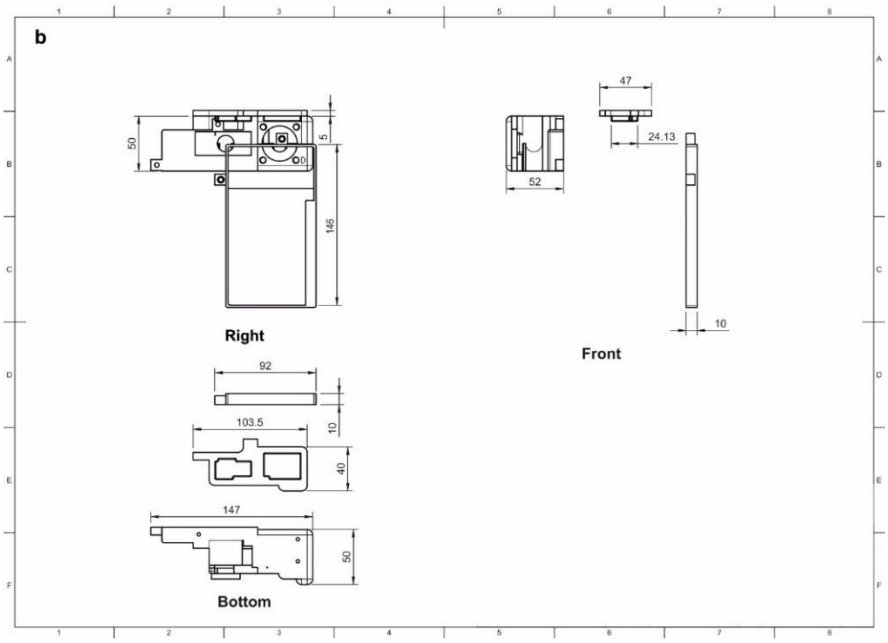

Supplementary Figure S3. Annotated 3D printing design for housing a portable Raman system (values are given in centimetres). (a). Right side and top views of the housing without amendments for D-Eye accessories, in wireframe with visible edges only visual style, with external walls and apertures annotated with dimensions. Perspective view also shown in shaded with visible edges only visual style **(b).** Right, bottom and front views of the housing along with the lid and D-Eye holder attachments, in wireframe with visible edges only visual style. (**Right).** Smart phone holder in front of the housing and the lid on top, showing the screw holes on the left-hand side of the housing and holder to join the two components. Once combined, the user can move the entire system by holding the phone. **(Front).** All three components expanded, illustrating the height and width added to the portable housing when the EyeD system assembled. **(Bottom).** The base of the housing when amended for the D-Eye accessories, the lid with protrusions that match the open top sections of the housing to eliminate external light entering the system and the D-Eye phone holder.

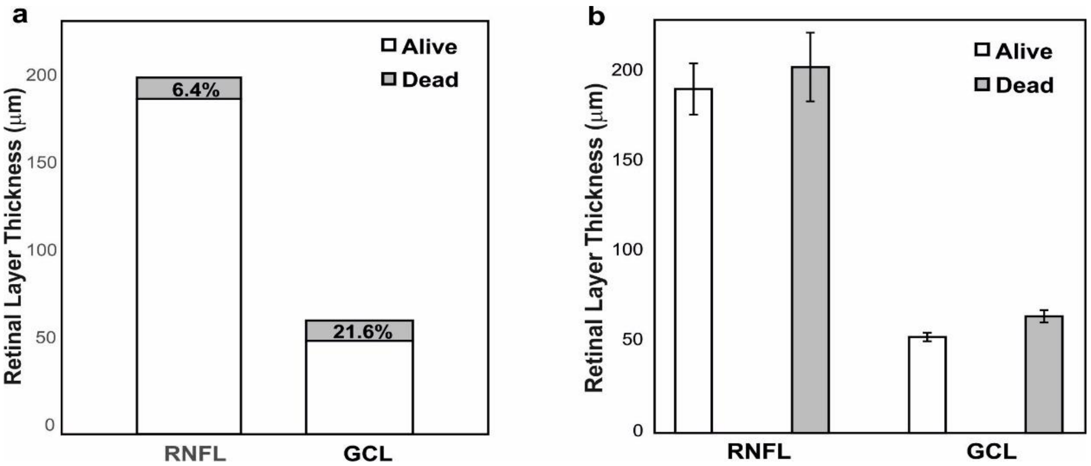

Supplementary Figure S4. Bar charts of OCT post-mortem and *in-vivo* porcine OCT images. a. The increase of RNFL and GCL after death and increase in RNFL and GCL thickness is consistent with a post-mortem cellular oedema and a larger increase of 21.6% measured in GCL (cell bodies), relative to 6.4% increase in RNFL. **b.** Increase in RNFL and GCL thickness following death.

Supplementary Table S3. Contribution from brain lipids in average spectra of retina samples for TBI group using NNLS fitting.

**Lipid**	#01	#02	#03	#04	#05	#06	#07	#08	#09	#10	#12		**Average**
**Cardiolipin**	0.83	0.85	0.79	0.88	0.84	0.84	0.83	0.85	0.82	0.87	0.84		0.84
Phosphatidylinositol	0	0	0	0	0	0	0	0	0	0	0		0
**Cholesterol**	0.016	0.015	0.009	0.007	0.015	0	0	0	0	0.009	0.012		0.00755
Cholesteryl ester	0	0	0	0	0	0	0	0	0	0	0		0
Galactocerebroside	0	0	0	0	0	0	0	0	0	0	0		0
Sphyingomyelin	0	0	0	0	0	0	0	0	0	0	0		0
**Cytochrome C**	0.78	0.81	0.85.0.77	0.78	0.83	0.81	0.75	0.77	0.81	0.82	0.84		0.8
Phosphatidylcholine	0	0	0	0	0	0	0	0	0	0	0		0
Phosphatidylserine	0	0	0	0	0	0	0	0	0	0	0		0
Sulfatide	0	0	0	0	0	0	0	0	0	0	0		0
**Ganlglioside**	0.51	0.41	0.45	0.51	0.25	0.37	0.45	0.42	0.41	0.4	0.49		0.42455
Phosphatidylethanolamine	0	0	0	0	0	0	0	0	0	0	0		0
Triacylglyceride	0	0	0	0	0	0	0	0	0	0	0		0
R^2	0.88	0.89	0.87	0.87	0.88	0.89	0.9	0.89	0.89	0.9	0.88		0.89

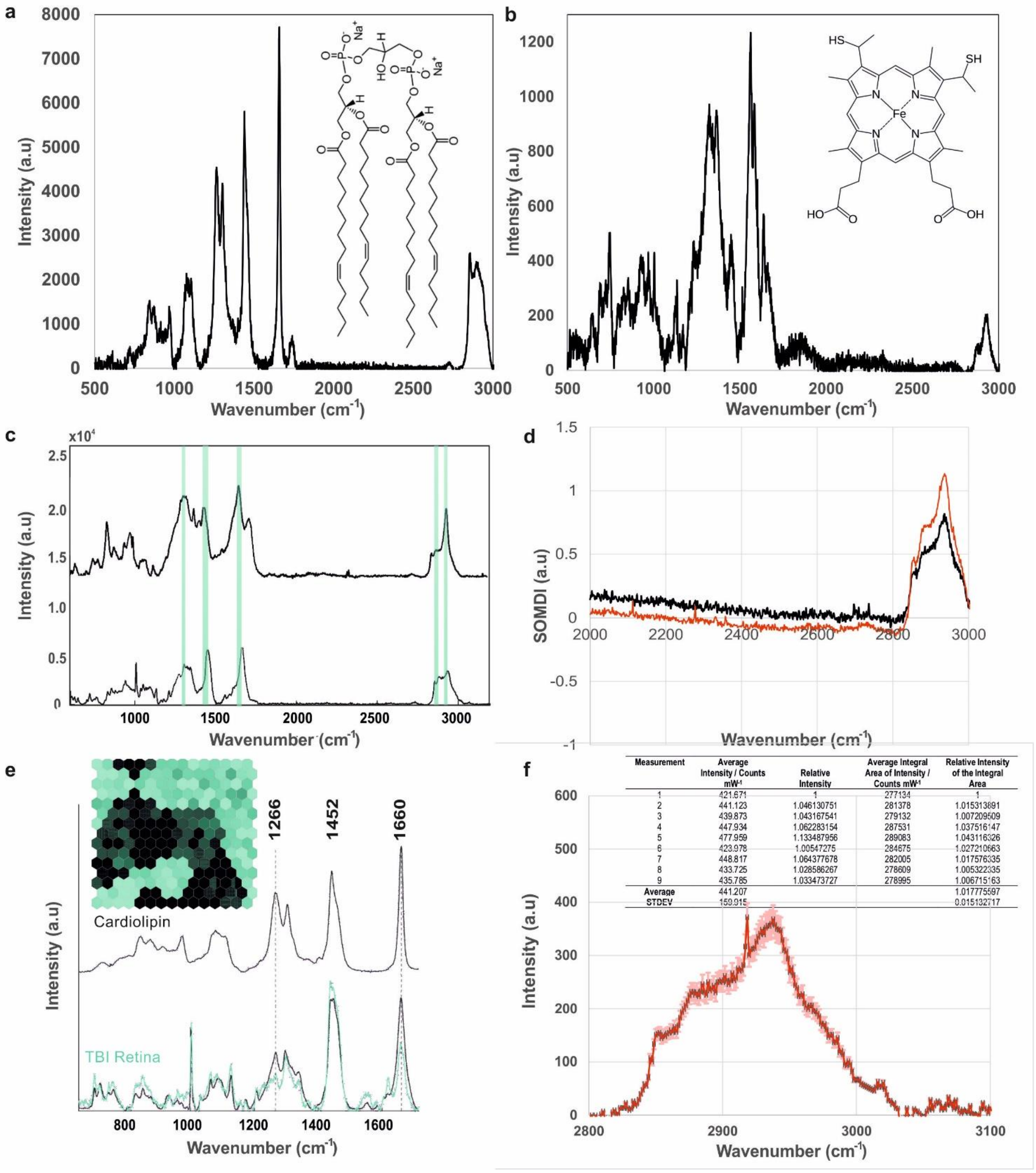

Measurement	Average Intensity / Counts mW-1	Relative Intensity	Average Integral Area of Intensity / Counts mW-1	Relative Intensity of the Integral Area
1	421.671	1	277134	1
2	441.123	1.046130751	281378	1.015313891
3	439.873	1.043167541	279132	1.007209509
4	447.934	1.062283154	287531	1.037516147
5	477.959	1.133487956	289083	1.043116326
6	423.978	1.00547275	284675	1.027210663
7	448.817	1.064377678	282005	1.017576335
8	433.725	1.028586267	278609	1.005322335
9	435.785	1.033473727	278995	1.006715163
Average	441.207			1.017775597
STDEV	159.915			0.015132717

Supplementary Figure S5. Fingerprinting Target Neuromarkers and Signal Reproducibility. Characteristic spectroscopic fingerprints of raw **(a)** Cardiolipin and **(b)** Cytochrome C and of measured from spiked porcine eye **(c)** Cardiolipin and Cytochrome C markers (Renishaw and EyeD) with specific bands highlighted at 1266cm^{-1}, 1452cm^{-1}, 1660cm^{-1} and the peaks at 2850 and 2930cm^{-1}, representative of the changes to relative lipid and protein composition as a result of TBI. **(a-b, inset)** The corresponding molecular structures yielding the characteristic spectra. **(d).** SOMDI extracted most prominent features of the high-wavenumber region, using the commercial Renishaw system (red) and the portable EyeD technology (black). **(e)** Comparative average Raman spectra of Cardiolipin to the measured from retina samples (green) of the *fingerprint region*, collected under the same experimental conditions, generating the SOM clustering **(inset)** for TBI (green) and control (black) groups. **(f)** Average Raman spectra collected from retina samples (n_{TBI}=39) of the high-wavenumber region using the EyeD. Shaded area outlines the standard deviation (STDEV) range. The reproducibility coefficient, derived based on the differences between measurements being normally distributed and the limits of agreement in a range within which 95% of future differences in establishing the mean and the standard deviation for representative measurements, exhibits good reproducibility with less than 8% variation of the signal intensity (reproducibility coefficient: STDEV $_{Average}$×2.8×100%=7.2%).

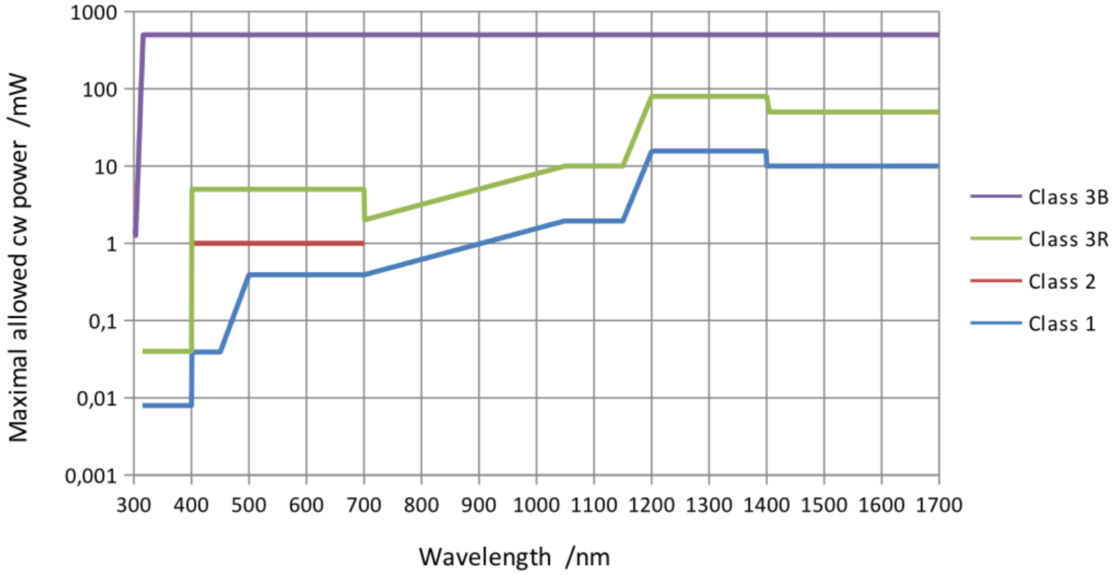

Supplementary Figure S6. Technical Specifications: Accessible emission limits for the maximal allowed powers for different laser classes according to the standard EN 60825-1:2014. The portable EyeD device uses a Conformité Européenne (CE) marked Class I laser conforming with EEA health & safety standards [635 nm class I Laser (Kingfisher International)], which guarantees eye safety. CE marking indicates conformity with European healthy and safety regulations, which leaves manufacturers at risk of fines or imprisonment if products are found to be non-compliant. Lasers are classified by wavelength and power into four classes and a few subclasses. The classifications categorize lasers according to their ability to produce damage in exposed people, from class 1 (no hazard during normal use) to class 4 (severe hazard for eyes and skin). There are two classification systems, the "old system" used before 2002, and the "revised system" being phased in since 2002. The latter reflects the greater knowledge of lasers that has been accumulated since the original classification system was devised and permits certain types of lasers to be recognized as having a lower hazard than was implied by their placement in the original classification system. The revised system is part of the new IEC 60825 standard, which from 2007, is also incorporated into the US-oriented ANSI Laser Safety Standard (ANSI Z136.1). Since then, labelling according to the revised system is accepted by the FDA on laser products imported into the US. The revised classification of a laser is based on the concept of accessible emission limits (AEL) that are defined for each laser class. This is usually a maximum power (in W) that can be emitted in a specified wavelength range and exposure time that passes through a specified aperture stop at a specified distance. It is the responsibility of the manufacturer to provide the correct classification of a laser, and to equip the laser with appropriate warning labels and safety measures as prescribed by the regulations. The developed portable EyeD system uses battery-powered 635 nm class I Laser [(Kingfisher International) at 0.39-0.63mW, which is well below the threshold for Class 1 laser ['BSI EN 60825-1, Safety of Laser Products. Part 1: Equipment Classification and Requirements. 2014'] and conforms to safety regulations as stipulated by IEC60825 standard, the US ANSI Laser Safety Standard (ANSI Z136.1) and European standard EN207.Per definition, a Class 1 laser is safe under all conditions of use. This means, maximum permissible exposure (MPE) cannot be exceeded when viewing a laser with the naked eye or with the aid of magnifying optics [IEC60825-1]). It is considered safe under all conditions of use with no safety concerns in eyes. Adopted from Ref. 79.

Supplementary Text S1. A Brief Overview of Ophthalmological Technologies for the Assessment of TBI:

1) Optical coherence tomography (OCT) allows structural assessment of the retina and optic nerve head. In the acute phase (<1 week after injury), there are no detectable changes. Late changes (1-2 weeks after injury) demonstrate neuronal loss and gliosis. OCT is very expensive and is only available in few specialised centres. It is operated and interpreted by trained ophthalmologists.
2) OCT angiography, a variant of the above, may show acute changes in blood flow, but there is only one published case report in TBI, so this is speculative. Current imaging devices have limited portability (being bulky desktop devices).
3) MRI spectroscopy provides the most directly comparable information, allowing *in-vivo* metabolomic assessment of the vitreous, which potentially reflects retinal metabolic changes, but requires an MRI scanner and can look directly at brain metabolic changes, whilst ophthalmic MRI spectroscopy has not been reported after TBI.
4) Advanced brain MR imaging (spectroscopy, functional MRI and Diffusion Tensor Imaging) is reported to measure and track changes after TBI but is only available in few highly specialised centres and the practical application suffers from significant variability in the techniques and lack of harmonised protocols.
5) Pupillometry is a functional assessment that demonstrates acute changes in optic nerve function after TBI in portable devices (although the only current binocular device is a desktop platform). This provides neurophysiological information, which may complement and not necessarily conflict with biological information from EyeTBI or similar test.
